# 01. Serum Bactericidal Activity Against Circulating and Reference Strains of Meningococcal Serogroup B in the United States: A Review of Meningococcal Serogroup B (MenB) Vaccines in Adolescents and Young Adults

**DOI:** 10.1093/ofid/ofab466.204

**Published:** 2021-12-04

**Authors:** Tamera Coyne-Beasley, Joseph Bocchini, Alejandro Cane, Cindy Burman, Maria J Tort, Jessica Presa

**Affiliations:** 1 University of Alabama at Birhmingham, Birmingham, Alabama; 2 Willis-Knighton Health System, Shreveport, Louisiana; 3 Pfizer Inc, Buenos Aires, Buenos Aires, Argentina; 4 Pfizer, Inc., Collegeville, Pennsylvania

## Abstract

**Background:**

US adolescents and young adults are at particular risk of invasive meningococcal disease (IMD). In 2018, menincococcal serogroup B was responsible for 36% of IMD cases in the US overall and for 66% of cases in adolescents and young adults. This age group is at high risk of IMD during outbreaks, which result in significant response-related costs. MenB vaccine efficacy against IMD relies on its ability to provide broad protection against diverse disease-causing strains. MenB-FHbp (Trumenba) and MenB-4C (Bexsero) are MenB vaccines licensed in the US as 2-dose series with an interval of 6 mo or 1 mo, respectively, recommended in healthy adolescents and young adults. We review available data on vaccine coverage of serogroup B strains.

**Methods:**

A literature review identified relevant information from peer-reviewed publications, congress presentations, and ClinicalTrials.gov. Previously presented but unpublished data from phase 2/3 studies were included.

**Results:**

After 2 MenB-FHbp doses, percentages of adolescents and young adults achieving serum bactericidal activity assay using human complement (hSBA) titers ≥1:8 were 79%–99% for 4 heterologous representative test strains and 71%–97% for 10 additional strains, confirming cross-protection against a diverse strain panel (**Figure 1**; unpublished data). These 14 heterologous strains collectively represent ~80% of disease-causing strains in the US and Europe. In a published study with limited sample size, 44%–78% of subjects had hSBA titers ≥1:8 against strains from 4 US college outbreaks after 2 MenB-FHbp doses. After 2 MenB-4C doses, percentages of 10–25-year-olds achieving hSBA titers ≥1:5 against 3 reference strains homologous to the vaccine antigen were 82%–93% (published data); 15%–100% of adolescents achieved hSBA titers ≥1:4 against a panel of 14 strains (unpublished data). Of college students who received 2 MenB-4C doses, 53%–93% achieved hSBA titers ≥1:4 against 5 US outbreak strains (4/5 strains had antigenic similarity to MenB-4C; published data).

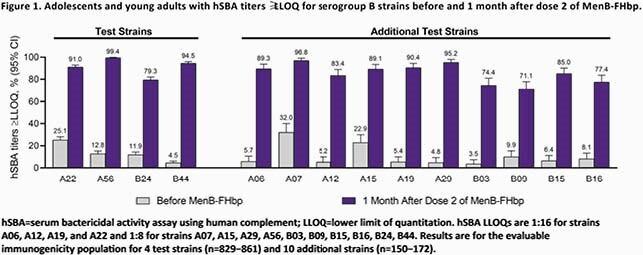

**Conclusion:**

MenB-FHbp and MenB-4C protect against various serogroup B strains. As for the breadth of coverage provided by these vaccines, available data show that MenB-FHbp elicits robust immune responses to a wide variety of disease-causing strains prevalent in the US (**Figure 2**).

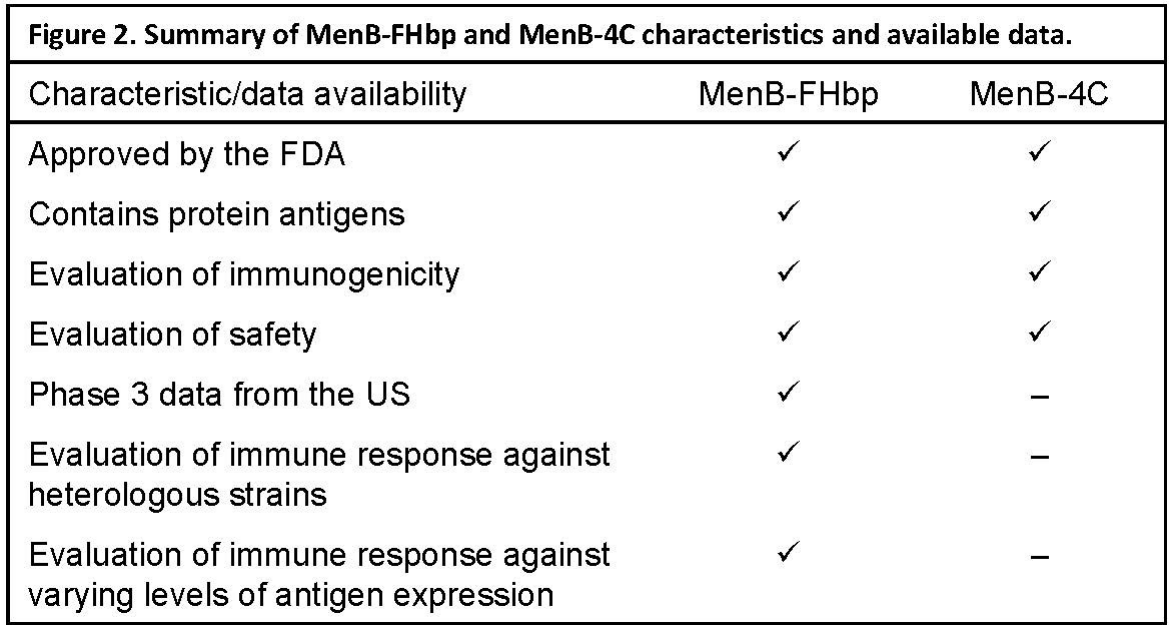

**Disclosures:**

**Tamera Coyne-Beasley, MD, MPH**, **Pfizer Inc and GlaxoSmithKline** (Advisor or Review Panel member) **Joseph Bocchini, MD**, **Pfizer Inc and Dynavax** (Advisor or Review Panel member) **Alejandro Cane, M.D.**, **Pfizer Inc** (Employee, Shareholder) **Cindy Burman, PharmD**, **Pfizer Inc** (Employee, Shareholder) **Maria J. Tort, PhD**, **Pfizer Inc** (Employee, Shareholder) **Jessica Presa, MD**, **Pfizer Inc** (Employee, Shareholder)

